# An allosteric role for receptor activity-modifying proteins in defining GPCR pharmacology

**DOI:** 10.1038/celldisc.2016.12

**Published:** 2016-05-17

**Authors:** Joseph J Gingell, John Simms, James Barwell, David R Poyner, Harriet A Watkins, Augen A Pioszak, Patrick M Sexton, Debbie L Hay

**Affiliations:** 1 School of Biological Sciences, University of Auckland, Auckland, New Zealand; 2 Maurice Wilkins Centre for Molecular Biodiscovery, University of Auckland, Auckland, New Zealand; 3 School of Life and Health Sciences, Aston University, Birmingham, UK; 4 Department of Biochemistry and Molecular Biology, University of Oklahoma Health Sciences Center, Oklahoma City, OK, USA; 5 Drug Discovery Biology and Department of Pharmacology, Monash Institute of Pharmaceutical Sciences, Monash University, Parkville, VIC, Australia

**Keywords:** Amylin, accessory protein, CGRP, G protein-coupled receptor, G protein-coupled receptor, RAMP

## Abstract

G protein-coupled receptors are allosteric proteins that control transmission of external signals to regulate cellular response. Although agonist binding promotes canonical G protein signalling transmitted through conformational changes, G protein-coupled receptors also interact with other proteins. These include other G protein-coupled receptors, other receptors and channels, regulatory proteins and receptor-modifying proteins, notably receptor activity-modifying proteins (RAMPs). RAMPs have at least 11 G protein-coupled receptor partners, including many class B G protein-coupled receptors. Prototypic is the calcitonin receptor, with altered ligand specificity when co-expressed with RAMPs. To gain molecular insight into the consequences of this protein–protein interaction, we combined molecular modelling with mutagenesis of the calcitonin receptor extracellular domain, assessed in ligand binding and functional assays. Although some calcitonin receptor residues are universally important for peptide interactions (calcitonin, amylin and calcitonin gene-related peptide) in calcitonin receptor alone or with receptor activity-modifying protein, others have RAMP-dependent effects, whereby mutations decreased amylin/calcitonin gene-related peptide potency substantially only when RAMP was present. Remarkably, the key residues were completely conserved between calcitonin receptor and AMY receptors, and between subtypes of AMY receptor that have different ligand preferences. Mutations at the interface between calcitonin receptor and RAMP affected ligand pharmacology in a RAMP-dependent manner, suggesting that RAMP may allosterically influence the calcitonin receptor conformation. Supporting this, molecular dynamics simulations suggested that the calcitonin receptor extracellular N-terminal domain is more flexible in the presence of receptor activity-modifying protein 1. Thus, RAMPs may act in an allosteric manner to generate a spectrum of unique calcitonin receptor conformational states, explaining the pharmacological preferences of calcitonin receptor-RAMP complexes. This provides novel insight into our understanding of G protein-coupled receptor-protein interaction that is likely broadly applicable for this receptor class.

## Introduction

G protein-coupled receptors (GPCRs) are essential proteins for cell signalling. They reside at the cell surface and interact with numerous factors [1]. These include extracellular constituents such as their ligands, integral membrane components including other GPCRs or GPCR accessory proteins and intracellular proteins, the most well known being G proteins. The propensity for GPCR function to be altered by allosteric mechanisms is substantial. Any factor that interacts with a GPCR has the potential to influence its conformation, and thus affecting its behaviour. This can include interactions with ligands or a second protomer in a receptor dimer [2]. With GPCRs being major targets for therapeutics, a profound understanding of the probable outcomes of such interactions on endogenous ligand and drug actions, both desired and undesirable, is needed.

The propensity for GPCRs to oligomerize offers opportunities to consider the impact of other membrane proteins on GPCR pharmacology [2]. The interpretation of such studies is often complicated by the inherent ability of each protomer to interact with ligand in its own right. However, there are GPCR accessory proteins that can offer novel insight into how membrane proteins alter GPCR pharmacology. A notable example of such proteins is the family of receptor activity-modifying proteins (RAMPs) [3].

There are three RAMPs, which each contains a structured extracellular N-terminal domain (ECD), a single transmembrane-spanning domain and a short intracellular C-terminus [4]. The key interactions between the RAMP and GPCR can reside in the N terminus or the transmembrane domain, depending on the specific GPCR partner for the RAMP [5, 6]. Such interactions affect GPCR properties such as altering pharmacology or signalling in a GPCR-specific manner. Eleven RAMP-interacting GPCRs have been identified to date with explicit pairings of only certain RAMPs with some of these GPCRs [3, 6–12], The largest cluster of GPCRs with which RAMPs interact reside within the ‘B’ class of peptide-binding GPCRs.

Like RAMPs, these GPCRs are characterized by a structured ECD of ~150 amino acids that forms a characteristic fold [13]. This GPCR ECD is the key domain for high ligand binding and interacts with the C-terminal portion of the natural peptide ligands for these receptors.

The best characterized interaction of RAMPs and a GPCR is the calcitonin receptor (CTR)-like receptor (CLR), where individual RAMPs co-expressed with CLR yield distinct receptors: calcitonin gene-related peptide (CGRP) receptors and adrenomedullin receptors. However, oligomerization of CLR with RAMPs is prerequisite for functional expression of the GPCR at the cell surface. In contrast, most other RAMP partners can be functionally expressed in the absence of RAMPs.

**Figure 1:**
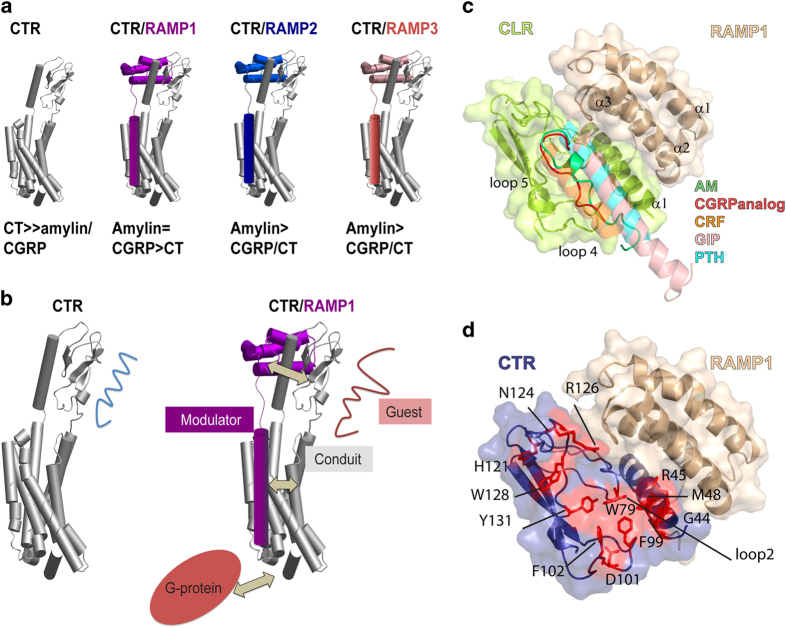
RAMPs modulate receptor function. (**a**) The CTR alone is a high-affinity receptor for CT, but in the presence of RAMP1, RAMP2 or RAMP3 is a high-affinity receptor for amylin. The RAMP1/CTR complex (AMY_1_ receptor) has high affinity for amylin and CGRP, while the RAMP2/CTR (AMY_2_ receptor) and RAMP3/CTR (AMY_3_ receptor), are high-affinity amylin receptors with lower affinity for related peptides. (**b**) Schematic representation showing how RAMPs are allosteric proteins that control GPCR behaviour. In particular, RAMPs alter the accessibility of the CTR extracellular domain-binding groove to amylin and CGRP. The affinity of CTR for these ligands is modified by the binding of RAMP. In CTR alone, amylin/CGRP interactions are suboptimal. RAMP alters the CTR-binding-site sufficiently to allow more optimal binding conditions. The diagram also shows how RAMPs have broader scope as allosteric proteins, with potential for altering GPCR properties via the transmembrane bundle and intracellular C-terminus, in addition to the ECD. G proteins are reported to influence RAMP-GPCR pharmacology, and RAMP-GPCR complexes may also influence G protein coupling. Thus, there is likely to be multidirectional allostery, which can explain the broad effects of RAMPs on numerous GPCRs, from pharmacology through to signalling and intracellular trafficking. (**c**) Crystal structure of the CGRP receptor ECD (CLR/RAMP1) with CGRPanalog ([D^31^, P^34^, F^35^]CGRP_27-37,_ red) bound and selected class B GPCR ligands: CRF (orange), adrenomedullin (AM) (green), GIP (salmon), PTH (cyan) superimposed. (**d**) Homology model of the AMY_1_ receptor ECD (CTR/RAMP1), with residues within the putative peptide-binding site that were selected for Ala mutation shown as red sticks.

The CTR is a notable example of a RAMP-interacting class B GPCR that is functionally expressed in the absence of RAMPs. CTR acts as a receptor for three hormones—calcitonin (CT), amylin and CGRP with a spectrum of affinities depending on its co-expression with RAMPs. Although CTR alone is a receptor for CT that is involved in bone homeostasis, CTR in complex with RAMP1-3 forms the AMY_1-3_ receptors (Figure 1a) [14], which are high-affinity receptors for amylin, a peptide involved in the regulation of food intake. The AMY_1_ receptor is also a high-affinity receptor for the neuropeptide CGRP [7, 15] (Figure 1a).

Recently, structures of the CGRP receptor (CLR+RAMP1) and the AM_1_ receptor (CLR+RAMP2) ECDs bound to a CGRP analogue ([D^31^, P^34^, F^35^]CGRP_27-37_) or AM_25-52_, respectively, were determined [16]. These structures revealed a unique mode of peptide binding without the α-helical structure observed in related class B GPCR ECD structures [17–21]. CGRP and AM instead are largely unstructured and feature a β-turn structure near the C-terminus enabling the C-terminal aromatic residue to form contacts with the RAMP (Figure 1). However, as RAMP co-expression is prerequisite for CLR expression and function, it provides only limited insight into how RAMPs modify the receptor to allow novel ligand pharmacology.

Unlike CLR, the CTR is functionally expressed at the cell surface in the absence of RAMPs. The well-defined pharmacology of CTR-RAMP complexes makes it an ideal system to probe how RAMP interaction enables novel GPCR function. We have investigated the role of CTR ECD residues in peptide–ligand interactions in the absence and presence of each RAMP. Mutagenesis, supported by modelling, reveals a common mode of peptide binding with the CLR-RAMP complexes but also suggests a role for allostery in RAMP-driven peptide selectivity via increasing conformational flexibility of the CTR ECD (Figure 1).

## Results

### Peptide interactions with the CTR ECD in the absence of RAMPs

Peptide binding to class B GPCR ECDs involves residues located within loops 2, 4 and 5 and the N-terminal α-helix of the ECD (Figure 1c) [22]. There are currently no structures of the CTR ECD available. Therefore, a homology model of the CTR ECD was produced based on the crystal structure of the CGRP receptor (Figure 1d). Ala mutagenesis was used to investigate the role of the CTR ECD residues that comprise the putative peptide-binding site (Figure 1d). The function of all the receptor mutants was characterized by cAMP assay, and cell surface expression measured by ELISA. Human CT (hCT) binding was measured at selected mutants through competition with ^125^I-hCT. We used the insert-negative form of CTR, which is named CT_(a)_ [14]. For all mutations, there was minimal impact on cell surface expression of the receptor (Supplementary Table S1).

**Figure 2:**
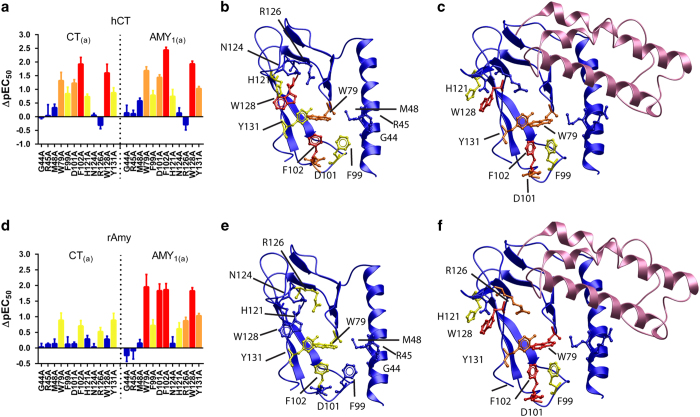
Effect of predicted binding-site residue mutations on CT_(a)_ or AMY_1(a)_ receptor function. (**a**–**c**) The effect of mutation on hCT-mediated responses at either the CT_(a)_ (**a**, left-hand panel; **b**) or AMY_1(a)_ (**a**, right-hand panel; **c**) receptor. (**d**–**f**) The effect of mutation on rAmy (rat amylin) -mediated responses at either the CT_(a)_ (**d**, left-hand panel; **e**) or AMY_1(a)_ (**d**, right-hand panel; **f**) receptor. Homology models of the isolated CTR (blue) (**b**, **e**) or the CTR/RAMP1 complex (**c**, **f**) (CTR, blue; RAMP1, pink). Mutated residues are displayed in x-stick and CPK and coloured according to the magnitude of effect of mutation on peptide function (red, >50-fold decreased potency; orange, 10–50-fold decreased potency; yellow, <10-fold decreased potency; and blue, not significantly different). Data points are mean±s.e.m., combined from four to six independent experiments, performed in duplicate or triplicate; and displayed as change in potency of the peptide (pEC_50_).

Ala mutation of selected residues in loops 2, 4 and 5 of the CTR ECD (W79, F99, D101, F102, H121, W128 and Y131) significantly reduced hCT potency and ^125^I-hCT binding (Figure 2a and b, Table 1 and Supplementary Figures S1 and S2A). In contrast, Ala mutation of residues G44, R45 and M48 within the N-terminal α-helix did not have any effect on hCT potency.

Having defined key residues for hCT binding in the CTR ECD, we next questioned whether lower affinity peptide ligands for CTR would require the same complement of residues. For amylin, W79A, F102A and Y131A had significantly reduced potency. R126A, which did not affect hCT potency, reduced amylin potency by approximately threefold (Figure 2d and e, Table 1 and Supplementary Figure S2B). Notably, two key residues for hCT (D101 and W128) did not affect amylin potency when mutated to Ala (Figure 2d, Table 1 and Supplementary Figure S2B). For CGRP, there were small reductions in potency at W79A, F99A, D101A, F102A, H121A, W128A and Y131A (Table 1). For both amylin and CGRP, the magnitude of effect of the mutation (apart from Y131A) was always smaller than that observed for hCT (Table 1 and Supplementary Figure S2A and B). Thus, while the pocket utilized by amylin and CGRP was substantially the same, only weak interactions were observed and these were confined to a subset of residues. The reduced impact of these mutations is consistent with the lower affinity of these ligands for CTR [14].

### RAMPs alter the role of CTR ECD residues in peptide–ligand binding

The AMY_1_ receptor complex (CTR/RAMP1) binds both amylin and CGRP with high affinity, while the human AMY_3_ receptor (CTR/RAMP3) is also a high-affinity receptor for amylin but has lower affinity for CGRP [23]. At the AMY_2_ receptor (CTR/RAMP2), there is only a weak enhancement in amylin and CGRP potency compared with the CTR, depending on the cell background and CTR splice variant [23–25]. Therefore, RAMP subtype can have a differential effect on pharmacology. The same set of residues initially tested at CTR expressed alone was also investigated in the presence of RAMP1. A subset of these was also tested with RAMP2 and RAMP3 to determine generalities in the mode of peptide binding to each subtype of AMY receptor.

Intriguingly, in the presence of RAMP1, the CTR residues required for high hCT potency at the CT_(a)_ were important for the enhanced potency of amylin and CGRP at the AMY_1(a)_ receptor. Mutation of W79, F99, D101, F102, H121, R126, W128 and Y131 to Ala each reduced potency of both peptides (Figure 2d and f, Table 2 and Supplementary Figure S2). ^125^I-hαCGRP binding was also abolished (Supplementary Figure S1D). In all cases, the magnitude of effect was greater at the AMY_1(a)_ receptor versus CT_(a)_. Notably, D101A and W128A, mutations that did not affect amylin potency at CTR alone, reduced both amylin and CGRP potency more than 50-fold in the presence of RAMP1 (Table 1 and Figure 2d and f). The mutations generally had similar effects at the AMY_2(a)_ and AMY_3(a)_ receptors (Supplementary Tables S2 and S3), although the magnitude of loss of peptide potency was generally less at AMY_2(a)_ compared with the other two amylin receptors, in parallel with the weaker induction of Amy potency seen with RAMP2. For CGRP, which has highest potency at the AMY_1(a)_ receptor, the D101A, F102A and W128A mutations had profound effects on potency in the presence of RAMP1, but not RAMP2 or RAMP3 (Supplementary Tables S2 and S3), suggesting that the allosteric modulation of these residues provides differentiation in CGRP interaction. R126A was without effect at AMY_2(a)_.

RAMP1 association with CTR, as measured by the presence of RAMP1 at the cell surface, was assessed for all CTR mutants (Supplementary Table S1). R126A resulted in lower RAMP1 cell surface expression of ~30%. This was matched by lower CTR expression to a similar degree, indicating that the reduced RAMP1 expression was most likely due to decreased CTR expression and thus less RAMP1 translocation to the cell surface, as oppose to diminished RAMP1 association with CTR. CTR cell surface expression was unchanged with any other mutant when expressed with RAMP1. We also examined RAMP2 expression with selected CTR mutants. Only R126A resulted in decreased expression, again paralleled with decreased CTR expression. We were unable to measure RAMP3 expression due to the absence of a suitably tagged version that behaves like wild-type (WT) RAMP3. Nevertheless we measured CTR mutant expression with RAMP3 and found no changes (Supplementary Table S1).

To determine whether the presence of RAMP affected the hCT binding site in CTR, hCT potency at the CTR mutations in the AMY_1(a)_ receptor was determined. The effects mirror those observed for CTR alone (Figure 2a and c and Supplementary Table S4), most likely because these responses are generated through CTR that is uncomplexed with RAMP, which is consistent with a population of ‘free’ CTRs [23].

### Modelling amylin and CGRP interactions with the AMY_1_ ECD

**Figure 3:**
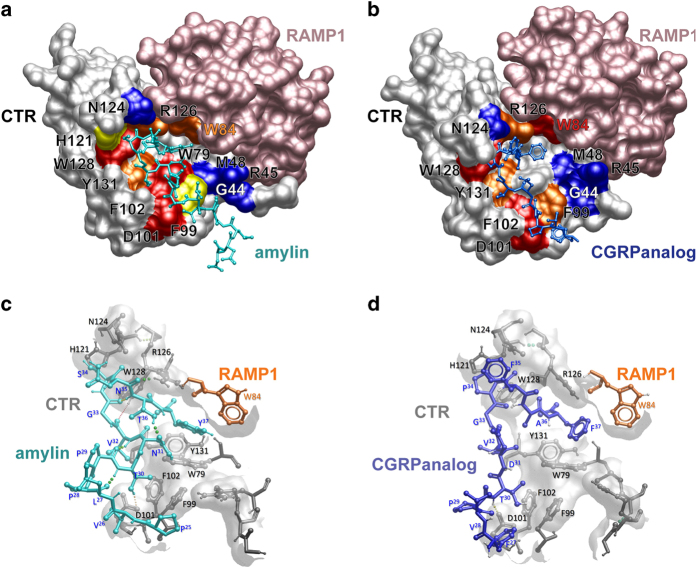
Homology models of the AMY_1_ receptor ECD (CTR, off-white surface; RAMP1, pink surface) in complex with rAmy (rat amylin, light blue) (**a**), or CGRPanalog [D^31^,P^34^, F^35^]CGRP_27-37_ (dark blue) (**b**), illustrating the predicted mode of peptide binding at the AMY_1_ receptor. The surfaces of key residues mutated in the current study are highlighted according to the magnitude of effect of mutation on peptide function (red, >50-fold decreased potency; orange, 10–50-fold decreased potency; yellow, <10-fold decreased potency; and blue, not significantly different). (**c** and **d**) illustrate how the peptides interact with the peptide-binding groove. Receptor residues are in off-white x-stick, RAMP1 residue W84 is in orange and the peptides are blue. Peptide amino acid numbers are displayed as superscript and receptor numbers in normal type. Hydrogen bonds are illustrated as coloured spheres. (**c**) rAmy (rat amylin). (**d**) CGRP analogue.

To help interpret our data, we modelled amylin and CGRP analogue interactions with the AMY_1_ receptor ECD using the structure of the CGRP analogue bound to the CGRP receptor ECD as a template (PDB ID: 4RWG) (Figure 3). There is a considerable sequence conservation between the two peptides (Supplementary Figure S3) and this is reflected by the binding modes predicted from the models. The proposed peptide-binding site is formed between loops 2, 3 and 5 of the CTR ECD and RAMP1 residue W84, with the N-terminal region of the (truncated) peptide located in proximity to loop 4. Both CGRP and amylin contain an aromatic residue at the C-terminus (Phe in CGRP, Tyr in amylin) and the hydrophobic interaction between the aromatic C-terminal residue of the peptide and the RAMP1 residue W84 is apparently conserved (Figure 3c and d). CTR residue D101 forms hydrogen bonds with T30 of the peptides in our models. Receptor residues W79, F99, F102 and Y131 that form the core of the binding site make hydrophobic interactions with the peptide (Figure 3c and d). Residues H121, R126 and W128 in loop 5 of the CTR ECD form contacts with the β-turn structure of the CGRP analogue (Figure 3d). Thus the models suggest that the key peptide interactions with the CLR ECD are conserved with the CTR ECD and therefore it is unclear what drives peptide selectivity between the CLR and the CTR.

### Residue differences between receptor sequences in the peptide-binding site do not contribute to peptide selectivity between CTR and CLR

There are, however, several residues within or in proximity to the peptide-binding site that differ between the CTR and CLR (Supplementary Figures S3A and S4). To investigate whether residue differences at these positions contribute to peptide selectivity, CTR residues at these positions were mutated to the equivalent residue in the CLR: G44T, P100Q, E123A, N124S and S129T. If these residues contributed to selectivity, the mutants would be expected to result in reductions in hCT and amylin potency because CLR has very low affinity for these ligands [14, 15]. At the CT_(a)_ and AMY_1(a)_ receptors, none of these mutants affected hCT, amylin or CGRP (Supplementary Table S5 and Supplementary Figure S4), suggesting that the residue differences between the CLR and CTR ECDs do not contribute to peptide selectivity between the two receptors.

### Mutation of interface residues between CTR and RAMP1 and dynamics of the complex

**Figure 4:**
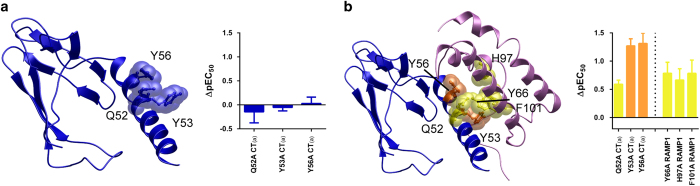
Effect of mutation on predicted CTR/RAMP1 interface residues. Homology model of the isolated CTR (blue) (**a**) or the CTR/RAMP1 complex (CTR, blue; RAMP1, pink) (**b**). Insets are graphs of the change in rAmy (rat amylin) cAMP potency (pEC_50_) at either the CT_(a)_ (**a**) or AMY_1(a)_ (**b**) receptors compared with WT. Mutated residues (Q52, Y53 and Y56 in CTR, and Y66, H97 and F101 in RAMP1) are displayed in x-stick and CPK and coloured according to the magnitude of effect of mutation on peptide function (orange, 10–50-fold decreased potency; yellow, <10-fold decreased potency; and blue, not significantly different). Data points are mean±s.e.m., combined from four to six independent experiments, performed in duplicate or triplicate.

The lack of any obvious mechanism for peptide specificity directly within the peptide-binding site, coupled with the differences observed in the role of residues D101 and W128 between the CTR and AMY_1_ receptor, led us to question whether RAMPs act allosterically to influence the role of residues within CTR. To test this, we hypothesized that altering the RAMP-CTR interface could affect amylin and CGRP potency. Amino acid sequence alignment and modelling suggested that CTR had a similar interaction interface with RAMP1 to that observed for CLR, involving residues along the N-terminal helix of the ECD (Figures 1d and 4). Mutation of Q52, Y53 and Y56 to Ala did not affect hCT or amylin potency in CTR alone but resulted in significant decreases in amylin and CGRP potency at the AMY_1(a)_ receptor (Figure 4, Supplementary Figure S5 and Table 3). Q52A resulted in a modest reduction in potency, while larger effects were observed with Y53A and Y56A. Interestingly, these mutations substantially reduced ^125^I-CGRP binding, even though these residues are not part of the peptide-binding site (Figure 1c). Cell-surface expression was unaffected with all of these mutations, suggesting that any disruption in the interface between CTR and RAMP was not sufficient to affect the amount of receptor at the cell surface (Supplementary Table S1). A similar pattern of RAMP-dependency was observed with these mutations at RAMP3 but not RAMP2-based receptors (Supplementary Tables S2 and S3).

We next examined the effects of interface mutations in RAMP1 by mutating the conserved RAMP residues Y66, H97 and F101. Each mutation resulted in significant decreases in amylin and CGRP potency and CGRP binding (Figure 4, Supplementary Figures S1 and S5 and Table 3). There was a greater reduction in CGRP potency in comparison to amylin, suggesting that disruption of the interface can have ligand-dependent effects. However, there were also significant reductions in cell surface expression, which may partially account for the loss of amylin and CGRP potency (Supplementary Table S6), though not the differences between the peptides.

**Figure 5:**
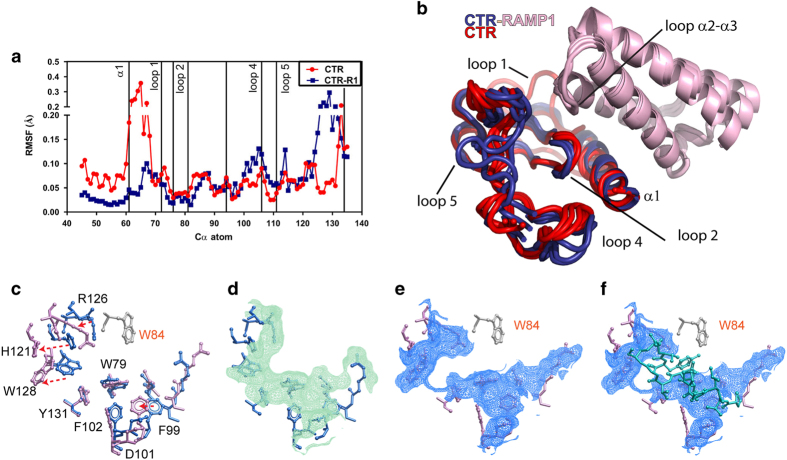
The RAMP1 ECD influences the dynamics of the CTR ECD. (**a**) Comparison of RMSF of the principle eigenvector for CTR alone and in complex with RAMP1. (**b**) Movement of the cα atoms of the CTR alone (red) with CTR (blue) in complex with RAMP1 (pink) showing the midpoint and extreme fluctuations of the principle eigenvector. (**c**) X-stick representation of the key residues for peptide binding for the CTR alone (blue) or in the presence of RAMP1 (pink), with W84 of RAMP in off-white. Residues in loop 5 (H121, R126 and W128) and in loop 2 (F99, D101 and F102) are substantially displaced when RAMP1 interacts with the receptor, and this is consistent with the MD simulations. (**d**–**f**) display the reorganization of the surface of the peptide-binding pocket; (**d**) CTR alone; (**e**) CTR in the presence of RAMP1; and (**f**) CTR in the presence of RAMP1 displaying the modelled amylin peptide (blue).

To investigate whether RAMP1 influences the conformation of the CTR ECD, extended molecular dynamics simulations were performed. The association of RAMP1 with CTR produces significant changes in the structure and dynamics of the latter in our simulations (Figure 5).

With the exception of loop 1, during a 1 μs molecular dynamics simulation, the CTR structure is relatively stable in the absence of RAMP1, suggesting that the binding pocket has limited flexibility. By contrast, after association with RAMP1, loops 4 and 5 and particularly the C-terminus of the CTR model become much more dynamic (Figure 5). The C-terminus undergoes a complex, writhing movement leading to considerable displacement of residues from R126 to Y131 (Figure 5c). This was analysed by principle component analysis and it was found that the majority of the movement (60%) could be described by the first eigenvector (Supplementary Figure S6), although the same pattern was observed in all of the first ten eigenvectors (Supplementary Figure S6). The movement was maintained over the majority of the entire 1 μs simulation (Supplementary Figure S6). The movements in loops 4 and 5 are consistent with the modelling of peptide binding to the AMY_1(a)_ receptor, with a predicted lateral extension and closure of the binding pocket associated with movement of loop 5 residues that likely facilitates interaction between the peptide C-terminus and the RAMP1 residue (W84) (Figure 5c and d versus Figure 5e and f). Similarly, the orientation of residues in loop 4, which are critical for high peptide potency, are also altered in models of peptide-bound receptors, whereas the key loop 2 residue (W79) is effectively unaltered in the model of amylin binding (Figure 5c). Because of the dynamic changes in the C-terminus there does not seem to be a single stable structure for this part of the CTR model, at least during the course of the simulation. The mechanism for the change may be the close proximity of the helix 2/3 loop of RAMP1 to loop 5 of the CTR ECD. In particular, this destabilizes the structure of loop 5 and C-terminus leading to the increased flexibility seen in the simulation. This flexibility in turn may be exploited by amylin and CGRP, allowing them to form better contacts than can be made with CTR alone when it is in a rigid state.

## Discussion

GPCRs are natural allosteric proteins that propagate extracellular signals to modulate intracellular signalling. Many GPCRs exist in protein complexes, including as homo- and hetero-GPCR dimers/oligomers as well as forming other protein–protein interactions with other classes of receptors, and various scaffolding, trafficking and modulating proteins. Most of these interactions lead to altered receptor function through formation of novel binding interfaces or by allosterically modulating GPCR function [2]. Nonetheless, we have only very limited molecular understanding of how these protein–protein interactions change receptor function.

As exemplar GPCR modulating proteins, with at least 11 GPCR partners, and a broad array of effects from altering cellular trafficking to engendering novel ligand pharmacology [3, 6–12], understanding of RAMP action provides insight into the spectrum of effects arising from protein-GPCR interaction. In the current study, we demonstrate that RAMPs allosterically alter the CTR N-terminal domain to enable high-affinity binding to amylin and CGRP peptides.

Recent advances in structural biology have provided important insights into the allosteric mechanism linking agonist binding to G protein activation, with this allosteric transition being driven by ordered changes in conserved hydrogen-bonded polar networks within the GPCR, leading to thermodynamically reciprocal changes in conformation of the intracellular surface of the receptor and to the ligand-binding pocket [26–28]. Of note, relatively small changes in the binding pocket, as are observed in the β2-adrenoceptor, can link to large changes in the intracellular face of the receptor that interacts with effector G proteins [29].

Similarly, although there is only limited structural information available, the allosteric cooperativity between small molecule modulators and orthosteric ligands is thought to principally occur via coupled conformational changes, although other factors such as electrostatic attraction or repulsion can also influence the interaction of spatially linked ligands [30].

Intriguingly, in the current study, the allosteric effect of RAMP on the ECD appeared to be principally driven by increased entropy in key loop regions of the ECD, in particular loop 5 and to a lesser extent loop 4. This was evident in the molecular dynamic simulations, with a high degree of correlation between the predicted increased flexibility of residues in these loops and the altered position of these residues in models of amylin peptide docking (Figure 5). Such entropically driven allostery is consistent with our evolving understanding of protein–protein allostery, where a range of allosteric mechanisms are now recognized, including the well-studied coordinated, ordered alteration to binding sites, as occurs for GPCR-effector coupling, but also includes entropically driven changes that increase protein flexibility, as well as ordered to disordered transitions [31, 32]. Thus, it is apparent that binding interactions can be affected by the interchange between protein stability and flexibility in transforming protein conformational landscapes, and that global dynamics and not just local interactions can modify surface properties of the protein to allosterically regulate responses, as reviewed by [31–33].

The recent solution of crystal structures of the peptide bound ECD of the CLR and RAMP1 or RAMP2 revealed that CGRP and AM have a distinct binding mode to other class B peptides and have limited secondary structure in complex with the receptor/RAMP N-terminal domain complex; they form interactions principally with loop regions of the receptor ECD in addition to a single important contact with the RAMPs via the peptide C-terminus [16]. This contrasts with other class B receptor/ligand complexes where an extended peptide α-helix is observed that interacts with the receptor N-terminal α-helix, in addition to loop contacts, although there is overlap in positioning of the CGRP/AM peptides and CRF (Figure 1c). Unlike CTR, CLR requires RAMP co-expression to enable peptide binding. The CTR alone has high affinity for CT peptides but low affinity for amylin and CGRP. Mutagenesis revealed a conserved binding pocket for all 3 peptides in both CTR alone and when complexed with RAMP, but the relative contribution of residues was markedly different between CTR alone and with RAMP.

Of the residues impacting on peptide function only R126 contributed to amylin or CGRP function but not that of CT. R126 in the CTR/RAMP complex sits in close proximity to W84 (RAMP1) that may form an interaction with the peptide C-terminus for amylin and CGRP, and thus may contribute to the orientation of the peptide C-terminus to allow this interaction. The net contribution of the RAMP interaction is a reordering of residues in loop 5 that facilitates the peptide turn of amylin and CGRP and interaction of the C-terminus with the W84 of the RAMP, with reorientation of loop 4 residues facilitating interactions between the receptor ECD and the peptide (Figure 5). In particular, modelling of peptide binding to the receptor suggests that both amylin and CGRP form an H-bond between T30 of the peptide and D101 of the receptor, while V32 contacts a hydrophobic patch comprised of W79, F102 and Y131 (Figure 3c). An Ala scan of an amylin analogue supports our models, with T30 and V32 substitutions abolishing binding. Interestingly, mutation of C-terminal Y37 had little effect on binding [34].

While CGRP has high affinity and potency at both AMY_1_ and CGRP receptors, only the AMY_1_ receptor has high affinity for amylin. The key residues for CGRP binding to both receptors are conserved between CTR and CLR, and these residues are also critical for amylin binding to the AMY_1_ receptor. To try to understand the selectivity difference we also mutated the non-conserved residues between CTR and CLR, however, these were not important for peptide potency/selectivity. Thus, the basis for peptide selectivity between the CGRP and AMY_1_ receptor cannot be readily explained by non-conserved interactions and is difficult to rationalize purely based on the N-terminal domain structures of the receptor complexes.

Although it is difficult to rationalize the selectivity differences between amylin and CGRP at the CGRP receptor without further experimentation, our data provides potential insight into the basis of selectivity of different RAMP/CTR complexes for CGRP. The three AMY receptors have similar affinity for amylin but only the AMY_1_ receptor has high affinity for CGRP. For amylin, the magnitude of effect of individual amino acid mutations was similar for different CTR/RAMP complexes, implying a similar allosteric effect on key residues to enable higher affinity binding. In contrast, the magnitude of effect on CGRP potency was greater for three of the residues, W128, D101 and F102 and the differential impact of these residues on CGRP interaction likely accounts for the higher potency of CGRP for the CTR/RAMP1 complex. Our previous studies using RAMP1/RAMP2 chimeras revealed that the short intracellular C-terminus of the RAMP plays a critical role in the higher potency of CGRP for AMY_1_ versus AMY_2_ receptors [35]. Thus, long-range allosteric effects are also important for the observed peptide interactions with the receptor ECD. Mutation of the interface between CTR and RAMP1 had more dramatic effects on CGRP than on amylin potency, suggesting that these long-range allosteric effects nonetheless are, at least in part, transmitted via the extracellular RAMP/CTR interface. Previous work has implicated the RAMP C-terminus in AMY receptor-effector coupling, particularly for G protein-dependent signalling [36], and it is therefore likely that it is the ternary complex of the receptor with effector protein that allosterically differentiates between amylin and CGRP binding. For CGRP and AM signalling, efficiency of G protein coupling (at least for Gs) is dependent upon the intracellular accessory protein, receptor component protein [37]. It is plausible that this difference could contribute to the selectivity difference for amylin between CGRP and AMY_1_ receptors.

RAMPs acting allosterically to determine CTR ligand selectivity is further supported by examining how RAMPs alter the conformation of the related CLR as evident in crystal structures of the CGRP analogue-bound CLR:RAMP1 and AM-bound CLR:RAMP2 ECD complexes [16]. A molecular morph between the two structures was generated to assess how RAMPs alter CLR conformation (Supplementary Movie S1). Although the morph does not represent a true physical pathway between conformational states, it is nonetheless useful for visualizing conformational change. The morph highlights movement of CLR α1 as well as shifts in several side chains on CLR α1 at the interface with the RAMPs. These changes appear to be propagated to the peptide-binding site in CLR resulting in subtle, yet likely significant changes to the shape of the peptide-binding site. RAMP1 and -2 clearly elicit different conformational states of CLR, which likely contributes to selectivity for CGRP or AM. It is conceivable that binding of RAMPs to the CTR N-terminal α-helix induces or stabilizes conformations of CTR that changes the peptide-binding site to better accommodate amylin and CGRP.

This study reveals that entropically driven allosteric effects have a role to play in GPCR based, protein–protein interactions. Furthermore, selectivity differences between different RAMP-CTR complexes for CGRP appeared to be driven by long-range allosteric interactions that are likely to involve interaction of intracellular effector proteins with the C-terminal domain of RAMPs, but that are nonetheless transmitted, at least in part, via the extracellular interface between the RAMP and the receptor ECD (Figure 1b). This work provides novel insight into how protein interactions modulate GPCR function.

## Materials and Methods

### Peptides

Rat amylin and hCT were purchased from American Peptide; human αCGRP was made in-house [38] or purchased from American Peptide. Peptides were made up as 1 mm stock solutions with ultrapure water and stored in siliconized microfuge tubes at −20 °C. Radioiodination of hCT was performed by adding 200* *μCi of NaI (PerkinElmer, Waltham, MA, USA) to 20 μg of hCT and incubating with an iodination bead (Pierce) for 5 min. I^125^ labelled hCT was then purified using reverse-phase HPLC, using a C18 column (Phenomenex, Torrance, CA, USA). ^125-^I-CGRP was purchased from PerkinElmer. The pharmacology of both radioligands was confirmed (Supplementary Figure S1).

### Plasmids

The double HA-tagged CTR construct used was generated as previously described [39]. This is the insert-negative form of the receptor and is referred to as hCT_(a)_, to comply with International Union of Pharmacology guidelines [40]. Myc tagged RAMP1 and untagged RAMP3 constructs were used for AMY receptor experiments. A RAMP2 construct with an N-terminal FLAG tag [41] was used for AMY_2(a)_ receptor experiments.

### Receptor mutagenesis

Mutagenesis was performed based on the Quick-change II method as previously described [42] with the following modifications. Primers were purchased from Integrated DNA Technologies Ltd (Coralville, IA, USA). Kapa HiFi readymix (Kapa Biosystems, Wilmington, MA, USA) or Primestar HS enzymes (TaKaRa Bio, Kyoto, Japan) were substituted for *Pfu* polymerase. RAMP1 constructs Y66A, H97A and F101A were produced as previously described [43].

### Cell culture

Cos-7 and HEK293S cells were cultured using DMEM high-glucose media (Invitrogen, Thermo Fisher, Waltham, MA, USA) supplemented with 8% fetal bovine serum (Moregate Biotech, Hamilton, New Zealand) in a humidified incubator at 37 °C, 5% CO_2_. Cells were seeded at a density of 1.5×10^5^–2.0×10^5^ cells per ml in 96-well plates. For ^125^I-hαCGRP experiments Cos-7 cells were seeded at a density of 7.5×10^5^ cells per ml in 24-well plates. Cells were transfected using polyethylenimine after 24 h and were grown for an additional 48 h before assaying as described previously [42]. Equal quantities of CTR and RAMP were used.

### cAMP assay

cAMP assays were performed as previously described [44] with the following modifications. After stimulation the reaction was stopped by removing media and adding 50 μl per well of ice-cold absolute ethanol. Plates were stored at −30 °C for up to 4 days before the measurement of cAMP was performed. The ethanol was allowed to evaporate and 40 (50 μl for HEK293S) of lysis buffer was added to each well before shaking for 15 min.

### Radioligand binding assay

Competitive radioligand binding assay was performed in whole cells. For ^125^I-hCT experiments, Cos-7 cells were plated and transfected in 96-well plates as per cAMP assay, for ^125^I-hαCGRP experiments Cos-7 cells were plated and transfected in 24-well plates. After 48 h, cells were washed with binding buffer (DMEM with 0.1% BSA), then incubated with binding buffer containing ~50 pm ^125^I-hCT or ^125^I-hαCGRP and increasing concentrations of unlabelled competitor peptide for 1 h at 37 °C. Cells were washed with ice-cold PBS pH 7.4 and lysed in 0.2 m NaOH. Radioactivity was measured using a Wizard2 gamma counter (PerkinElmer). Non-specific binding was determined using 1 μm unlabelled hCT or hαCGRP; total binding was established in the absence of a competitor peptide.

### ELISA

Cell-surface expression was determined by ELISA and performed as previously described [42]. Briefly, cells were plated and transfected as per cAMP assay. Cell-surface expression of receptor components was detected using mouse anti HA monoclonal antibody 1:2000 (Covance, Princeton, NJ, USA; MMS-101P), anti-myc 1:250 (Merck Millipore, Billerica, MA, USA; 9E10) or anti-FLAG 1:1000 (Sigma-Aldrich, St Louis, MO, USA; F1804-1G) to detect the tagged receptors. Goat anti-mouse biotin conjugate (Sigma-Aldrich) and Streptavidin HRP polymer (Sigma-Aldrich) were used to detect the antibodies using SigmaFast *o*-phenylenediamine dihydrochloride tablets (Sigma-Aldrich). Data were normalized to WT receptor transfected cells as 100% (either CTR with empty vector or RAMP) and empty vector transfected cells as 0%. Statistical significance was achieved if the 95% confidence interval did not include 100%.

### Homology modelling of CTR and AMY receptor ECDs

CTR and CLR full-length sequences were aligned using Tcoffee [45]. A multiple template design was used to generate the CTR ECD. CLR ECD, chain A from 3N7S, was used as a template structure for CTR ECD. The first loop domain of chain A (3N7S) is not elucidated. Therefore, the loop domain of 3N7S (chain B) was used as the template for this loop domain in the CTR ECD. Modeller9v8 [46] was used to generate 500 models. The models were ranked by the Modeller9v8 energy objective function. The top 10 structures were retained and the stereochemical quality was assessed by PROCHECKv3.5.4. [47]. On the basis of overall and residue-by-residue geometry a structure was selected. The ProPka program [48] via the PDBQPR server [49] was used to assign the protonation states of the titratable groups in CTR ECD, using the CHARMM parameters set at pH 7.0. The CHARMM (c35b3) module Screened Coulomb Potentials Implicit Solvent Model was used to minimize the model. 100 steps of steepest descent were conducted followed by adopted basis Newton-Raphson minimization until convergence was met.

To model CGRP and rat amylin bound to the AMY receptor, a similar approach was used to that for the modelling of the receptor without ligand, but the structure of a CGRP analogue bound to the CGRP receptor ECD was used as a template (PDB ID: 4RWG). This was mutated in-silico to give the structures of either human CGRP or amylin. Subsequent steps were as described above. PDB files are available as Supplementary Material (ctr_ecd; ctr_r1; ctr-ramp1-cgrpmut; ctr-ramp1-ramy).

### Molecular dynamics

Homology models of the ECD of the CTR alone and the CTR ECD docked to the RAMP1 ECD were based on the CLR/RAMP1 ECD structure 3N7R using a Clustal Omega alignment. With Modeller v9.10, 1 000 models were generated and scored using OPUS_PSP. The best scoring model was visually inspected before minimization using the Amber99sb ILDN force-field as implemented in GROMACS. For MD simulations, the minimized protein was placed centrally in a charge neutral cubic box (*x*=10 nm) consisting of TIP3 water molecules, 150 mm NaCl and simulated for 1 μs. Principal component analysis of the resulting trajectories was used to identify the main eigenvectors and the root mean square fluctuation calculated.

### Data analysis and statistics

Data for cAMP accumulation assay and radioligand binding were fitted to a three-parameter logistic equation in Prism 6 (Graphpad Software, San Diego, CA, USA) to determine the EC_50_ or IC_50_, after first using an F-test to determine whether the Hill-slope differed from 1. Statistical significance was determined through unpaired *t*-test. In each experiment cAMP data were normalized to the fitted minimum and maximum of the WT curve on each assay plate.

## Figures and Tables

**Table 1:** Summary of cAMP assay pEC_50_ values for CTR ECD alanine mutants at the CT_(a)_ receptor when stimulated with hCT, rAmy (rat amylin) or hαCGRP

*CT_(a)_*	*hCT*			*rAmy*			*hαCGRP*		
*Mutant*	*pEC_50_ WT*	*pEC_50_ mutant*	*Fold shift*	*pEC_50_ WT*	*pEC_50_ mutant*	*Fold shift*	*pEC_50_ WT*	*pEC_50_ mutant*	*Fold shift*
G44A	9.77±0.15 (3)	9.80±0.12 (3)		8.06±0.19 (4)	8.03±0.19 (4)		—	—	
R45A	9.51±0.19 (4)	9.47±0.32 (4)		7.87±0.10 (4)	7.75±0.10 (4)		—	—	
M48A	9.38±0.18 (5)	9.04±0.21 (5)		7.96±0.08 (4)	7.83±0.25 (4)		—	—	
W79A	9.49±0.28 (4)	8.17±0.14 (4)**	21	8.26±0.15 (6)	7.37±0.20 (6)**	8	6.97±0.08 (4)	6.35±0.16 (4)*	4
F99A	9.49±0.23 (5)	8.64±0.12 (5)*	7	8.15±0.13 (5)	8.03±0.16 (5)		7.06±0.15 (4)	6.63±0.10 (4)	
D101A	9.84±0.07 (5)	8.61±0.13 (5)***	17	8.17±0.04 (6)	8.04± 0.04 (6)		7.14±0.15 (6)	6.51±0.04 (6)**	4
F102A	9.51±0.16 (7)	7.44±0.16 (7)***	118	8.40±0.14 (5)	7.70±0.23 (5)*	5	6.99±0.08 (4)	6.30±0.06 (4)***	5
H121A	9.90±0.06 (5)	9.13±0.09 (5)***	6	8.17±0.17 (5)	7.97±0.14 (5)		7.26±0.19 (4)	6.58±0.04 (4)*	5
N124A	9.83±0.31 (3)	9.61±0.38 (3)		8.28±0.15 (4)	8.24±0.19 (3)		—	—	
R126A	9.26±0.07 (5)	9.57±0.17 (5)		8.64±0.19 (5)	8.12±0.11 (5)**	3	7.79±0.16 (5)	7.68±0.13 (5)	
W128A	9.80±0.24 (6)	7.96±0.06 (6)***	69	8.31±0.10 (5)	8.03±0.07 (5)		7.20±0.13 (6)	6.74±0.19 (6)	
Y131A	9.37±0.25 (5)	8.57±0.16 (5)*	6	8.40±0.19 (4)	7.51±0.29 (4)*	8	7.16±0.14 (4)	6.51±0.11 (4)*	5

Data are mean±s.e.m. Number of independent experiments indicated in parentheses. *P<0.05; **P<0.01; ***P<0.001 versus WT by unpaired *t*-test.

**Table 2:** Summary of cAMP assay pEC_50_ values for CTR ECD alanine mutants at the AMY_1(a)_ receptor (CT_(a)_/RAMP1) when stimulated with rAmy (rat amylin) or hαCGRP

*AMY_1(a)_*	*rAmy*			*hαCGRP*		
*Mutant*	*pEC_50_ WT*	*pEC_50_ mutant*	*Fold shift*	*pEC_50_ WT*	*pEC_50_ mutant*	*Fold shift*
G44A	9.42±0.33 (5)	9.65±0.35 (5)		9.98±0.06 (3)	10.13±0.16 (3)	
R45A	9.35±0.29 (5)	9.44±0.25 (5)		9.69±0.33 (3)	9.67±0.31 (3)	
M48A	9.51±0.21 (4)	9.35±0.13 (4)		9.52±0.26 (4)	9.22±0.32 (4)	
W79A	9.84±0.25 (6)	7.88±0.27 (6)***	91	9.43±0.25 (6)	7.46±0.25 (6)***	93
F99A	9.90±0.08 (5)	9.18±0.23 (5)*	5	10.19±0.12 (5)	9.04±0.26 (5)**	14
D101A	10.05±0.18 (6)	8.22±0.15 (6)***	68	9.74±0.28 (6)	6.90±0.17 (6)***	692
F102A	9.67±0.24 (5)	7.81±0.09 (5)***	72	9.74±0.18 (4)	6.79±0.19 (4)***	891
H121A	9.73±0.18 (5)	9.12±0.09 (5)*	4	9.62±0.15 (5)	8.60±0.14 (5)**	11
N124A	9.74 ±0.19 (4)	9.58±0.19		9.31±0.26 (5)	9.02±0.13 (5)	
R126A	9.35±0.28 (4)	8.48±0.18 (4)*	7	9.48±0.34 (4)	8.37±0.17 (4)*	13
W128A	9.90±0.14 (4)	8.00±0.18 (4)***	79	9.84±0.26 (4)	7.26±0.16 (4)***	380
Y131A	9.74±0.28 (4)	8.47±0.26 (4)*	19	10.11±0.18 (4)	8.64±0.29 (4)**	30

Data are mean±s.e.m. Number of independent experiments indicated in parentheses. *P<0.05; **P<0.01; ***P<0.001 versus WT by unpaired *t*-test.

**Table 3:** Summary of cAMP assay pEC_50_ values for ECD alanine mutants of either CT_(a)_ or RAMP1 at the predicted interface between these proteins in the CT_(a)_ and AMY_1(a)_ (CT_(a)_ /RAMP1) receptors when stimulated with hCT, rAmy (rat amylin) or hαCGRP

	*hCT*			*rAmy*		
*Mutant*	*pEC_50_ WT*	*pEC_50_ mutant*	*Fold Shift*	*pEC_50_ WT*	*pEC_50_ mutant*	*Fold Shift*
Q52A CT_(a)_	9.48±0.21 (5)	9.33±0.23 (5)	—	8.02±0.20 (5)	8.17±0.09 (5)	—
Y53A CT_(a)_	9.63±0.29 (5)	9.52±0.11 (5)	—	8.00±0.07 (4)	8.06±0.11 (4)	—
Y56A CT_(a)_	10.02±0.19 (4)	10.05±0.12 (4)	—	8.68±0.15 (5)	8.66±0.13 (5)	—
	***hαCGRP***			***rAmy***		
Q52A AMY_1(a)_	9.62±0.17 (4)	8.95±0.13 (4)*	5	9.66±0.12 (4)	9.07±0.09 (4)**	4
Y53A AMY_1(a)_	9.49±0.18 (4)	7.90±0.11 (4)***	39	9.52±0.22 (5)	8.25±0.20 (5)**	19
Y56A AMY_1(a)_	9.71±0.22 (6)	7.97±0.21 (6)***	55	9.78±0.18 (6)	8.47±0.17 (6)***	20
Y66A RAMP1	9.91±0.17 (4)	8.20±0.18 (4)***	51	9.67±0.21(4)	8.88 ±0.03 (4)**	6
H97A RAMP1	9.86±0.18 (4)	8.82±0.14 (4)**	11	9.67±0.21 (4)	9.00±0.14 (4)*	5
F101A RAMP1	9.82±0.11 (4)	8.49±0.15 (4)***	21	9.67±0.21 (4)	8.89±0.10 (4)*	6

Data are mean±s.e.m. Number of independent experiments indicated in parentheses. *P<0.05;**P<0.01; ***P<0.001 versus WT by unpaired *t*-test.
