# An allosteric role for receptor activity-modifying proteins in defining GPCR pharmacology

**DOI:** 10.1038/celldisc.2016.20

**Published:** 2016-06-21

**Authors:** Joseph J Gingell, John Simms, James Barwell, David R Poyner, Harriet A Watkins, Augen A Pioszak, Patrick M Sexton, Debbie L Hay

**Affiliations:** 1 School of Biological Sciences, University of Auckland, Auckland, New Zealand; 2 Maurice Wilkins Centre for Molecular Biodiscovery, University of Auckland, Auckland, New Zealand; 3 School of Life and Health Sciences, Aston University, Birmingham, UK; 4 Department of Biochemistry and Molecular Biology, University of Oklahoma Health Sciences Center, Oklahoma City, OK, USA; 5 Drug Discovery Biology and Department of Pharmacology, Monash Institute of Pharmaceutical Sciences, Monash University, Parkville, VIC, Australia

**Correction to:** *Cell Discovery* (2016) **2**, 16012; doi:10.1038/celldisc.2016.12; published online 17 May 2016

During web production, there was an error in Supplementary information: Supplementary Material was omitted. The files are now installed in the online version of the paper. 

We apologize for any inconvenience that may have been caused by this error.

