# Supplementary material for: An allosteric role for receptor activity-modifying proteins in defining GPCR pharmacology
Source: Cell Discov. 2016 May 17;2:16012–. doi: 10.1038/celldisc.2016.12 (PMC4869360; doi:10.1038/celldisc.2016.12)
Supplement: Supplementary Figure S1 [file celldisc201612-s1.pdf]

Supplementary Figure S1. (A) Displacement of ^{125}I-hCT by hCT and rAmy in whole cells transfected with $CT_{(a)}$. Data are mean ± SEM of 2-3 independent experiments. Data are normalized to the maximum specific binding observed for each peptide. (B) Displacement of ^{125}I-hαCGRP by hαCGRP, hCT and rAmy in whole cells transfected with $AMY_{1(a)}$. Data are mean ± SEM of 3 independent experiments. Data are normalized to the maximum specific binding observed for each peptide. (C) Specific binding of ^{125}I-hCT to selected CTR mutants at the $CT_{(a)}$, data are mean ± SEM of 3-5 independent experiments performed in whole cells. (D) Specific binding of ^{125}I-hαCGRP to selected CTR mutants at the $AMY_{1(a)}$ receptor, data are mean ± SEM of 3 independent experiments performed in whole cells. (E) Specific binding of ^{125}I-hαCGRP selected RAMP1 mutants at the $AMY_{1(a)}$ receptor, data are mean ± SEM of 3 independent experiments performed in whole cells. For C, D and E statistical significance (*) was achieved if the 95% confidence interval did not include 100%.

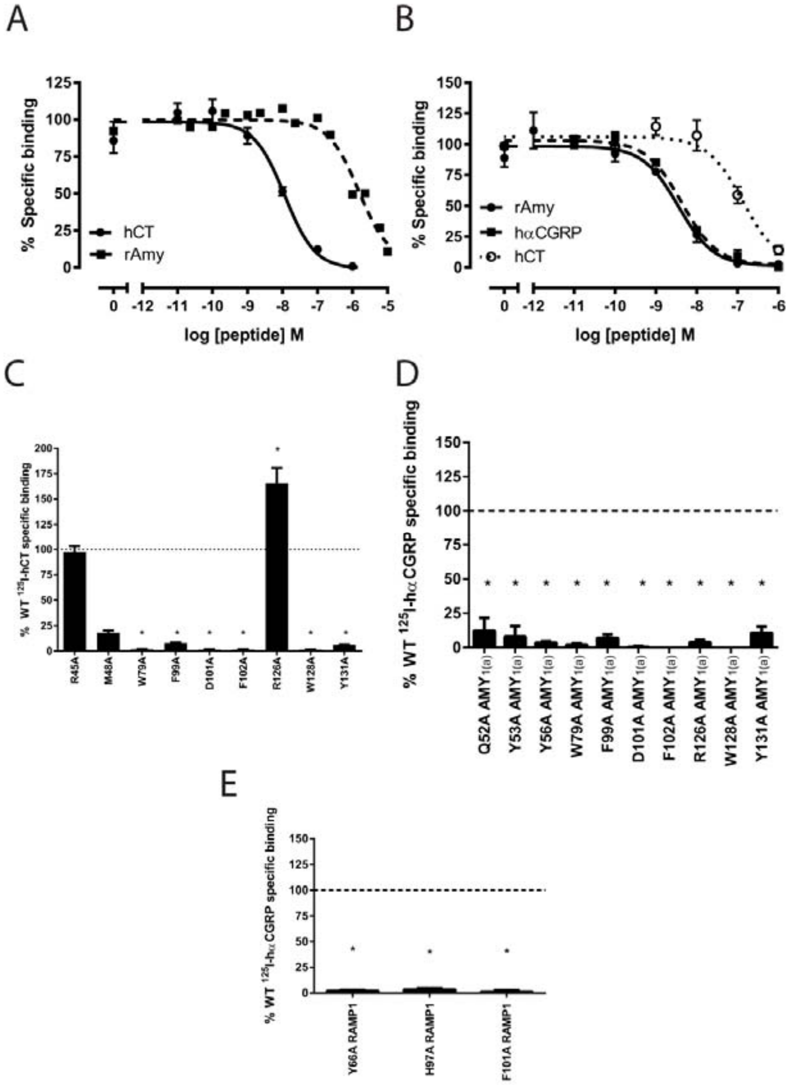
A
% Specific binding
hCT
rAmy
log [peptide] M
B
% Specific binding
rAmy
hαCGRP
hCT
log [peptide] M
C
% WT 125I-hCT specific binding
R45A
M48A
W79A
F99A
D101A
F102A
R126A
W128A
Y131A
D
% WT 125I-hαCGRP specific binding
Q52A AMY1(a)
Y53A AMY1(a)
Y56A AMY1(a)
W79A AMY1(a)
F99A AMY1(a)
D101A AMY1(a)
F102A AMY1(a)
R126A AMY1(a)
W128A AMY1(a)
Y131A AMY1(a)
E
% WT 125I-hαCGRP specific binding
Y66A RAMP1
H97A RAMP1
F101A RAMP1
