# Supplementary material for: An allosteric role for receptor activity-modifying proteins in defining GPCR pharmacology
Source: Cell Discov. 2016 May 17;2:16012–. doi: 10.1038/celldisc.2016.12 (PMC4869360; doi:10.1038/celldisc.2016.12)
Supplement: Supplementary Figure S2 [file celldisc201612-s2.pdf]

Supplementary Figure S2. cAMP accumulation of CTR mutants in response to hCT (A), or rAmy (B) at the $CT_{(a)}$, or rAmy at the $AMY_{1(a)}$ receptor (C), transiently expressed in Cos-7 cells. Data are combined from 4-6 independent experiments, performed in duplicate or triplicate; data points are mean ± SEM.

A

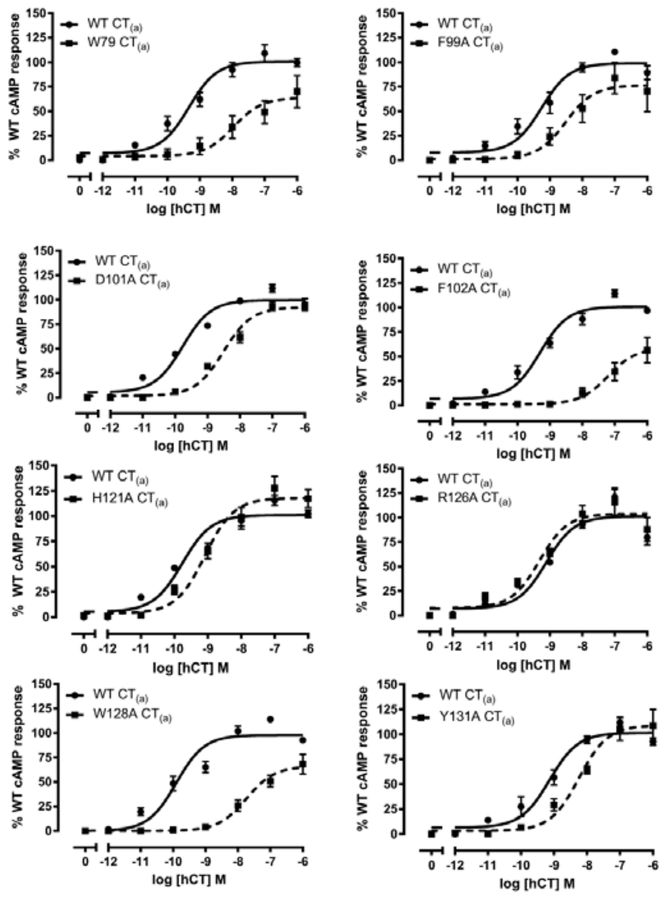

B

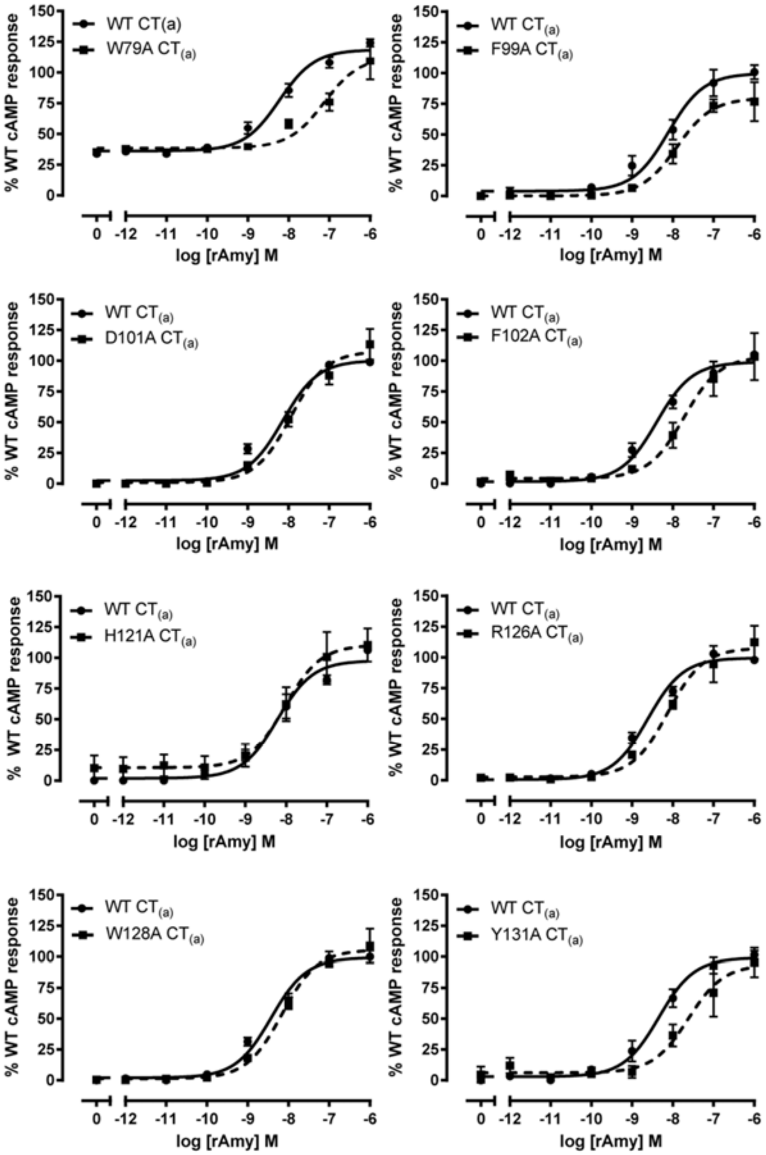
WT CT(a)
W79A CT(a)
F99A CT(a)
D101A CT(a)
F102A CT(a)
H121A CT(a)
R126A CT(a)
W128A CT(a)
Y131A CT(a)
% WT cAMP response
log [rAmy] M

C

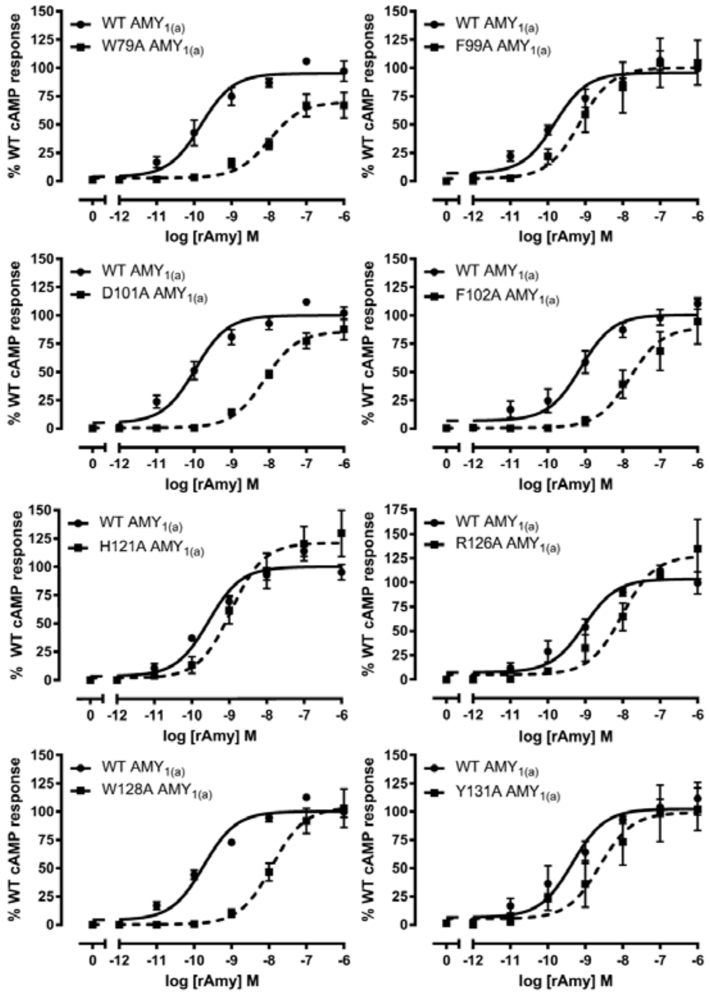

WT AMY1(a)
W79A AMY1(a)
F99A AMY1(a)
D101A AMY1(a)
F102A AMY1(a)
H121A AMY1(a)
R126A AMY1(a)
W128A AMY1(a)
Y131A AMY1(a)
% WT cAMP response
log [rAmy] M
