# Supplementary material for: An allosteric role for receptor activity-modifying proteins in defining GPCR pharmacology
Source: Cell Discov. 2016 May 17;2:16012–. doi: 10.1038/celldisc.2016.12 (PMC4869360; doi:10.1038/celldisc.2016.12)

Supplementary Figure S3. (A) Amino acid sequence alignment of CTR and CLR across several species generated using the Geneious align function in Geneious v 8.1. Residues highlighted in red boxes were changed in human CTR to the human CLR equivalent. (B) Amino acid sequence alignment of CT family peptides, conserved C-terminal residues are highlighted in red boxes.

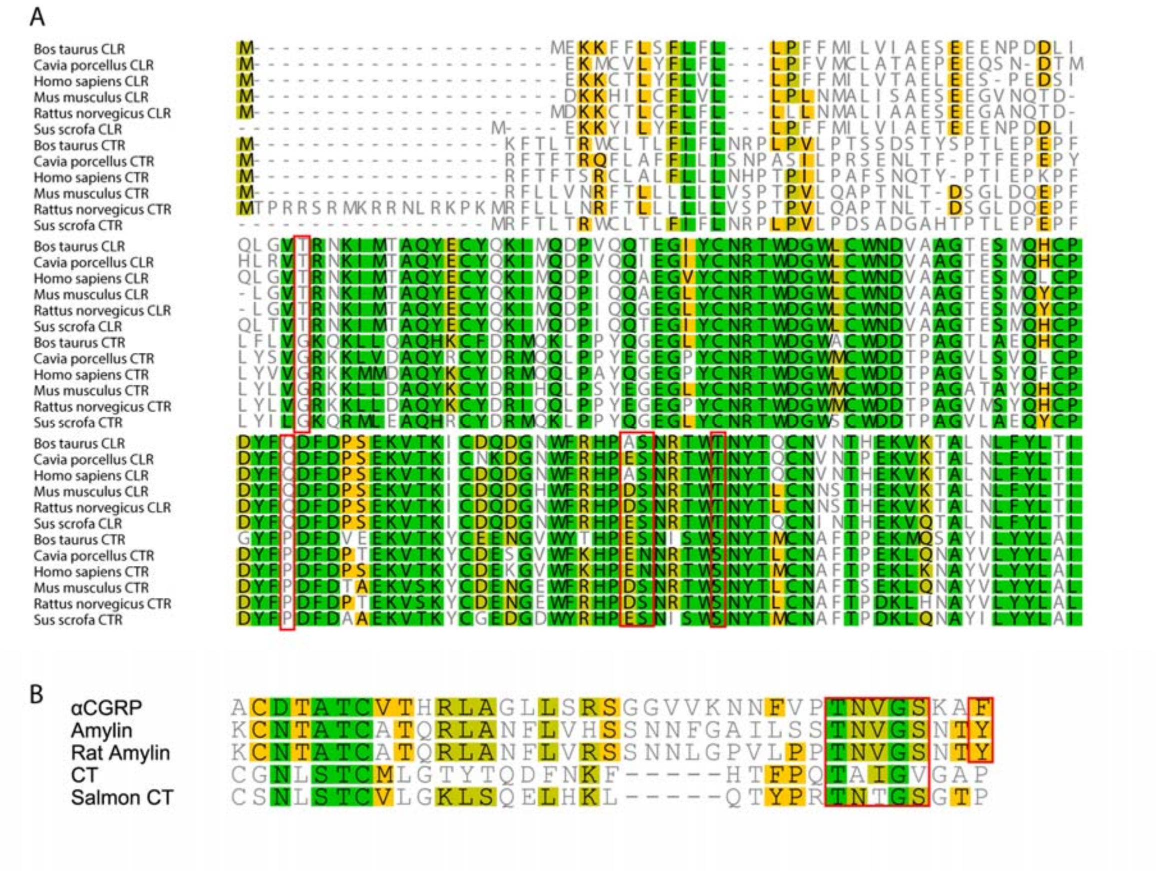

Supplement: Supplementary Figure S3 [file celldisc201612-s3.pdf]
