# Supplementary material for: An allosteric role for receptor activity-modifying proteins in defining GPCR pharmacology
Source: Cell Discov. 2016 May 17;2:16012–. doi: 10.1038/celldisc.2016.12 (PMC4869360; doi:10.1038/celldisc.2016.12)

**Supplementary Figure S4.** Homology model of the CTR ECD, showing the position where CTR residues were swapped for the CLR equivalent (blue x-stick). None of the mutations had any effect on amylin or CGRP responses.

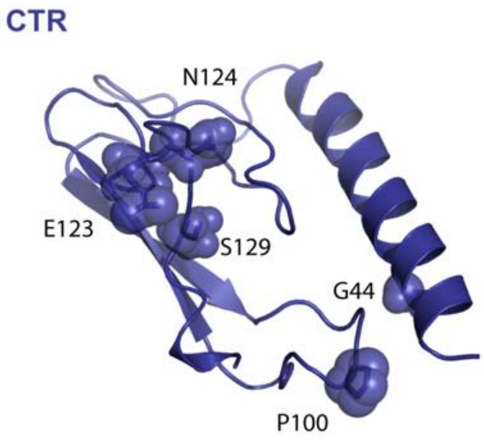

Supplement: Supplementary Figure S4 [file celldisc201612-s4.pdf]
