# Supplementary material for: An allosteric role for receptor activity-modifying proteins in defining GPCR pharmacology
Source: Cell Discov. 2016 May 17;2:16012–. doi: 10.1038/celldisc.2016.12 (PMC4869360; doi:10.1038/celldisc.2016.12)

Supplementary Figure S5. cAMP accumulation of CTR and RAMP1 ECD interface mutants in response to rAmy (A) at the $CT_{(a)}$ and $AMY_{1(a)}$ receptors and hαCGRP (C) at the AMY1(a) receptor in Cos-7 cells. Data are combined normalized from 3-5 independent experiments, performed in duplicate or triplicate, data points are mean ± SEM. (B, D) Position of interface residues in homology models of the AMY_1 receptor ECD, mutated residues are illustrated as sticks, with the effect of the mutation in the full-length receptor from the cAMP data summarized in the accompanying legend.

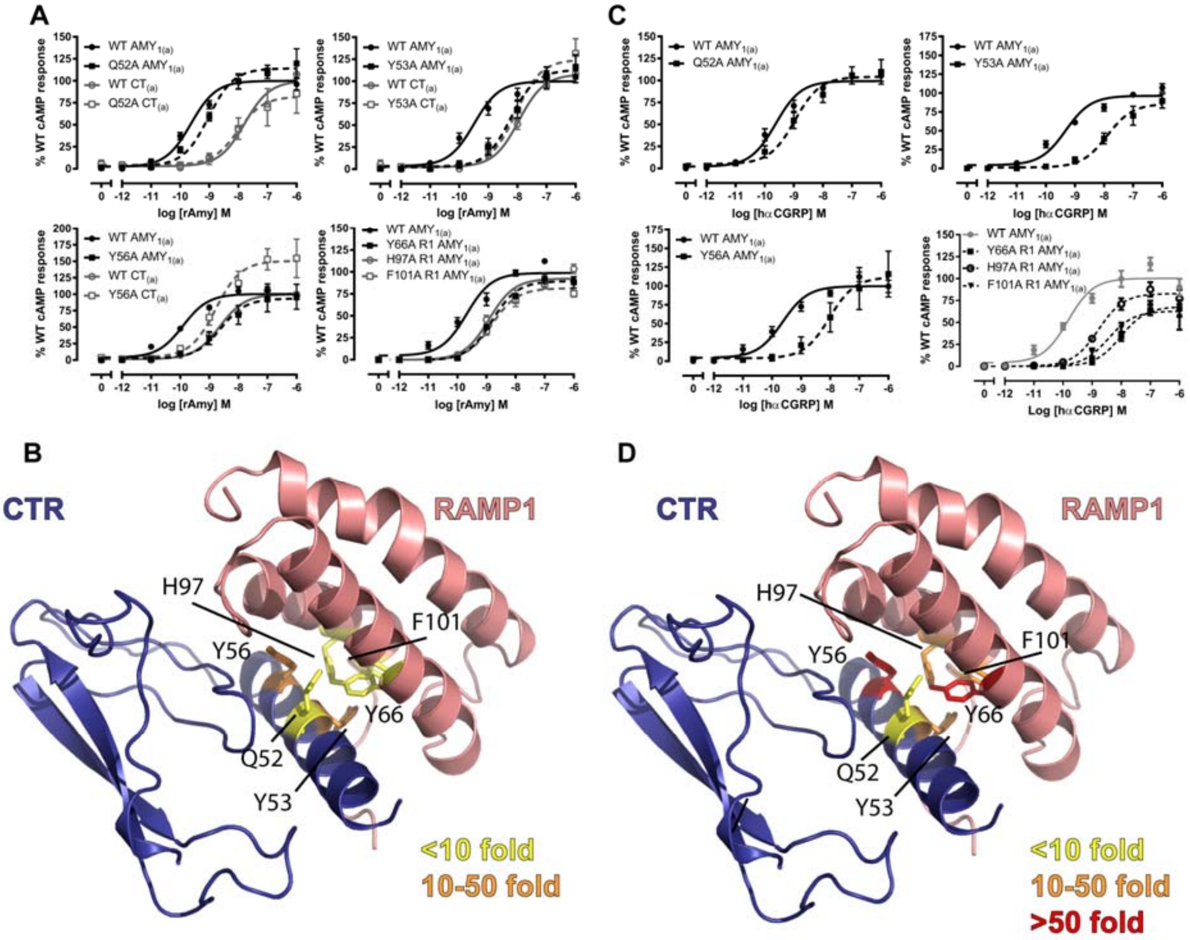

Supplement: Supplementary Figure S5 [file celldisc201612-s5.pdf]
