# Supplementary material for: An allosteric role for receptor activity-modifying proteins in defining GPCR pharmacology
Source: Cell Discov. 2016 May 17;2:16012–. doi: 10.1038/celldisc.2016.12 (PMC4869360; doi:10.1038/celldisc.2016.12)

Supplementary Figure S6. Comparison between the RMSF of the first 10 eigenvectors (EV) arising from principle component analysis of the movement of the extracellular domains of CTR in complex with RAMP1 (A), CTR alone (B) and RAMP1 in the presence of CTR (C) during a 1μs molecular dynamics simulation (C). In (D), the RMSF of the CTR-RAMP1 of the complex is shown against time over the 1μs simulation.

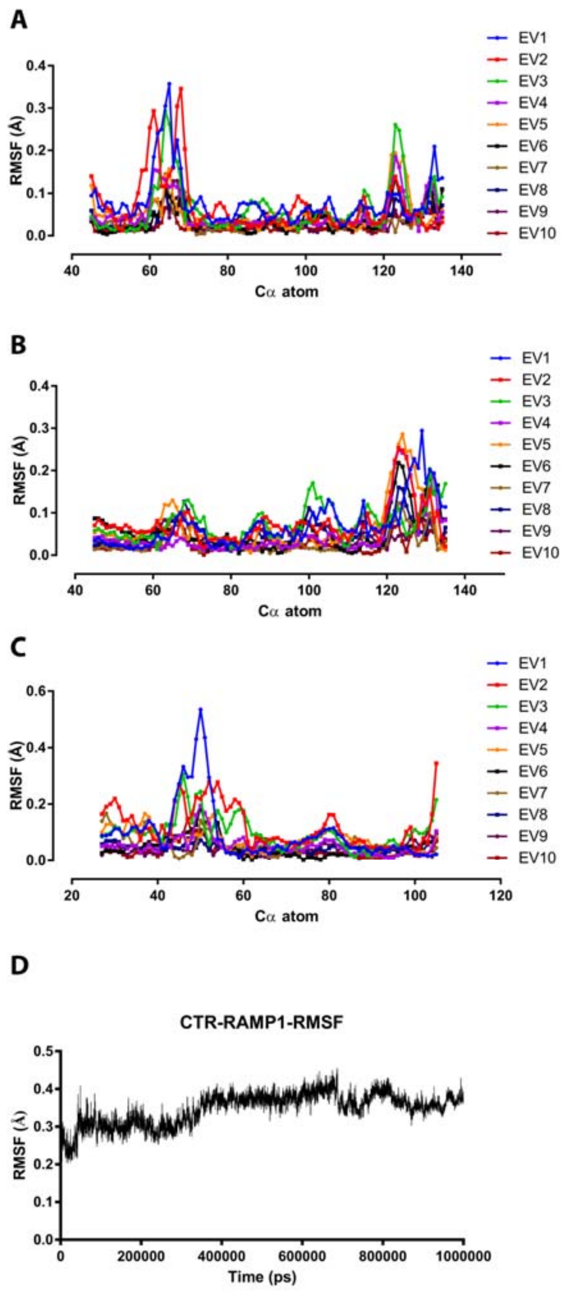

Supplement: Supplementary Figure S6 [file celldisc201612-s6.pdf]
