# Supplementary material for: An allosteric role for receptor activity-modifying proteins in defining GPCR pharmacology
Source: Cell Discov. 2016 May 17;2:16012–. doi: 10.1038/celldisc.2016.12 (PMC4869360; doi:10.1038/celldisc.2016.12)
Supplement: Supplementary Table S1 [file celldisc201612-s7.pdf]

Supplementary Table S1. Summary of cell surface expression for CTR ECD mutants at the $CT_{(a)}$, $AMY_{1(a)}$, $AMY_{2(a)}$ and $AMY_{3(a)}$ receptors. Data are mean ± SEM, the number of independent experiments is indicated in parentheses. Statistical significance (*) was achieved if the 95% confidence interval of the mean did not include 100 %.

	**$CT_{(a)}$ % WT**	**$AMY_{1(a)}$ %WT**		**$AMY_{2(a)}$ % WT**		**$AMY_{3(a)}$ % WT**
**Mutant**	**Anti-HA**	**Anti-HA**	**Anti-myc**	**Anti-HA**	**Anti-FLAG**	**Anti-HA**
**G44A**	95.3 ± 4.4 (3)	94.9 ± 14.3 (3)	94.9 ± 9.7 (3)	-	-	-
**G44T**	99.6 ± 23.8 (4)	117.2 ± 3.8 (4)	106.8 ± 8.8 (4)	-	-	-
**R45A**	116.2 ± 11.9 (3)	95.1 ± 10.8 (3)	94.4 ± 4.3 (3)	-	-	-
**M48A**	79.2 ± 10.3 (3)	61.7 ± 11.5 (3)	58.7 ± 5.0 (3) *	-	-	-
**Q52A**	99.9 ± 11.5 (3)	111.4 ± 19.5 (3)	123.4 ± 21.1 (3)	108.7 ± 14.8 (3)	79.3 ± 0.55 (3)	128.3 ± 12.4 (4)
**Y53A**	124.1 ± 11.5 (3)	102.0 ± 14.0 (4)	94.9 ± 10.9 (4)	111.0 ± 10.0 (3)	91.4 ± 12.6 (3)	130.4 ± 12.8 (3)
**Y56A**	106.7 ± 22.0 (4)	121.4 ± 8.4 (5)	109.4 ± 26.4 (5)	122.0 ± 5.7 (3)	95.1 ± 12.0 (3)	120.8 ± 11.1 (3)
**W79A**	77.2 ± 5.8 (5) *	90.9 ± 18.6 (4)	79.7 ± 19.1 (4)	97.5 ± 7.6 (3)	97.1 ± 16.4 (3)	112.1 ± 13.3 (4)
**F99A**	119.8 ± 10.8 (4)	147.7 ± 32.0 (5)	190.1 ± 14.1 (4)	118.1 ± 12.1 (3)	96.5 ± 16.7 (3)	129.3 ± 23.9 (4)
**P100Q**	141.8 ± 21.7 (3)	99.7 ± 5.8 (3)	111.3 ± 11.2 (3)	-	-	--
**D101A**	132.1 ± 14.9 (4)	121.9 ± 12.4 (5)	152.6 ± 32.7 (5)	103.2 ± 2.3 (3)	104.8 ± 2.2 (3)	122.8 ± 8.3(3)
**F102A**	128.0 ± 13.7 (4)	115.4 ± 17.2 (6)	117.9 ± 21.0 (6)	107.5 ± 5.2 (3)	101.7 ± 8.5 (3)	130.3 ± 27.4 (3)
**H121A**	96.4 ± 28.3 (4)	118.3 ± 12.9 (4)	102.2 ± 3.4 (4)	-	-	-
**E123A**	90.7 ± 13.6 (4)	95.0 ± 3.3 (3)	97.3 ± 15.6 (3)	-	-	-
**N124A**	115.6 ± 8.9 (4)	100.5 ± 2.6 (3)	111.1 ± 17.0 (3)	-	-	-
**N124S**	110.9 ± 7.1 (4)	105.9 ± 13.2 (3)	118.6 ± 10.1 (3)	-	-	-
**R126A**	83.8 ± 9.9 (3)	57.6 ± 15.6 (3)	70.0 ± 10.7 (3)	58.2 ± 14.9 (4)	54.5 ± 15.5 (4)	111.5 ± 12.4 (3)

**W128A**	95.0 ± 12.3 (4)	131.5 ± 20.8 (5)	161.7 ± 34.4 (4)	120.0 ± 0.7 (3)	118.1 ± 6.2 (3)	134.3 ± 17.1 (3)
**S129T**	136.8 ± 6.2 (4)	85.8 ± 12.5 (3)	100.0 ± 13.2 (3)	-	-	-
**Y131A**	107.4 ± 7.4 (5)	109.1 ± 24.0 (5)	112.7 ± 25.7 (4)	126.1 ± 13.8 (3)	112.6 ± 8.1 (3)	133.2 ± 15.6 (3)
