# Supplementary material for: An allosteric role for receptor activity-modifying proteins in defining GPCR pharmacology
Source: Cell Discov. 2016 May 17;2:16012–. doi: 10.1038/celldisc.2016.12 (PMC4869360; doi:10.1038/celldisc.2016.12)
Supplement: Supplementary Table S2 [file celldisc201612-s8.pdf]

Supplementary Table S2. Summary of cAMP assay pEC_{50} values for CTR ECD alanine mutants at the $AMY_{2(a)}$ receptor when stimulated with rAmy or hαCGRP in HEK293S cells. Data are mean ± SEM. Number of independent experiments indicated in parentheses. * $p<0.05$; ** $p<0.01$; *** $p<0.001$ versus WT by unpaired t-test.

**$AMY_{2(a)}$**	**rAmy**			**hαCGRP**		
**Mutant**	**pEC_{50} WT**	**pEC_{50} mutant**	**Fold shift**	**pEC_{50} WT**	**pEC_{50} mutant**	**Fold shift**
**Q52A**	8.57 ± 0.32 (5)	8.06 ± 0.21 (5)		-	-	
**Y53A**	8.69 ± 0.38 (4)	8.04 ± 0.22 (4)		-	-	
**Y56A**	8.57 ± 0.32 (5)	8.42 ± 0.21 (5)		-	-	
**W79A**	8.85 ± 0.10 (5)	7.39 ± 0.30 (5) **	29	7.95 ± 0.18 (3)	6.82 ± 0.24 (3) *	14
**F99A**	8.51 ± 0.26 (4)	7.57 ± 0.28 (4) *	9	7.95 ± 0.21 (4)	7.08 ± 0.17 (4) *	7
**D101A**	8.95 ± 0.09 (3)	8.04 ± 0.18 (3) *	8	8.57 ± 0.28 (3)	6.86 ± 0.02 (3) **	51
**F102A**	8.63 ± 0.22 (4)	7.26 ± 0.24 (4) **	23	7.98 ± 0.37 (3)	6.95 ± 0.44 (3)	11
**R126A**	8.92 ± 0.26 (4)	8.60 ± 0.28 (4)		8.02 ± 0.31 (3)	7.75 ± 0.12 (3)	
**W128A**	8.76 ± 0.24 (4)	7.30 ± 0.28 (4)**	29	8.45 ± 0.28 (4)	6.91 ± 0.24 (4) **	35
**Y131A**	8.65 ± 0.16 (5)	7.49 ± 0.17 (5) ***	15	8.33 ± 0.34 (4)	7.11 ± 0.04 (4) *	17
