# Supplementary material for: An allosteric role for receptor activity-modifying proteins in defining GPCR pharmacology
Source: Cell Discov. 2016 May 17;2:16012–. doi: 10.1038/celldisc.2016.12 (PMC4869360; doi:10.1038/celldisc.2016.12)
Supplement: Supplementary Table S3 [file celldisc201612-s9.pdf]

**Supplementary Table S3.** Summary of cAMP assay $pEC_{50}$ values for CTR ECD alanine mutants at the $AMY_{3(a)}$ receptor when stimulated with rAmy or hαCGRP. Data are mean ± SEM. Number of independent experiments indicated in parentheses. * p<0.05; ** p<0.01; *** p<0.001 versus WT by unpaired t-test.

| **$AMY_{3(a)}$** | **rAmy** | | | **hαCGRP** | | |
|---|---|---|---|---|---|---|
| **Mutant** | **$pEC_{50}$ WT** | **$pEC_{50}$ mutant** | **Fold shift** | **$pEC_{50}$ WT** | **$pEC_{50}$ mutant** | **Fold shift** |
| **Q52A** | 9.36 ± 0.09 (5) | 8.88 ± 0.21 (5) | | - | - | |
| **Y53A** | 9.51 ± 0.17 (5) | 8.59 ± 0.27 (5) * | 8 | - | - | |
| **Y56A** | 9.58 ± 0.18 (5) | 8.74 ± 0.22 (5) * | 7 | - | - | |
| **W79A** | 8.77 ± 0.30 (5) | 7.42 ± 0.15 (5) ** | 22 | 8.31 ± 0.24 (6) | 6.95 ± 0.12 (6) *** | 23 |
| **F99A** | 9.19 ± 0.26 (6) | 8.29 ± 0.17 (6) * | 8 | 8.09 ± 0.22 (5) | 6.96 ± 0.19 (5) ** | 14 |
| **D101A** | 9.24 ± 0.33 (4) | 8.14 ± 0.23 (4) * | 13 | 8.49 ± 0.14 (4) | 6.79 ± 0.04 (4) *** | 50 |
| **F102A** | 8.98 ± 0.32 (5) | 7.38 ± 0.15 (5) ** | 40 | 8.07 ± 0.30 (5) | 6.24 ± 0.19 (5) *** | 68 |
| **R126A** | 9.07 ± 0.29 (4) | 7.85 ± 0.39 (4) * | 17 | 8.14 ± 0.13 (4) | 7.38 ± 0.20 (4) * | 6 |
| **W128A** | 9.21 ± 0.22 (5) | 7.76 ± 0.15 (5) *** | 28 | 7.98 ± 0.26 (5) | 6.44 ± 0.17 (5) ** | 35 |
| **Y131A** | 9.11 ± 0.27 (5) | 8.22 ± 0.20 (5) * | 8 | 8.37 ± 0.22 (5) | 7.39 ± 0.19 (5) ** | 10 |
