# Supplementary material for: An allosteric role for receptor activity-modifying proteins in defining GPCR pharmacology
Source: Cell Discov. 2016 May 17;2:16012–. doi: 10.1038/celldisc.2016.12 (PMC4869360; doi:10.1038/celldisc.2016.12)
Supplement: Supplementary Table S4 [file celldisc201612-s10.pdf]

Supplementary Table S4.

Summary of cAMP assay pEC_{50} values for ECD alanine mutants of CTR in response to hCT at the $AMY_{1(a)}$ receptor in Cos-7 cells. Data are mean ± SEM, the number of independent experiments indicated in parentheses. * p<0.05, *** p<0.001 versus WT by unpaired t-test.

**$AMY_{1(a)}$**			
**Mutant**	**pEC_{50} WT**	**pEC_{50} mutant**	**Fold shift**
**G44A**	9.38 ± 0.14 (3)	9.24 ± 0.42 (3)	
**R45A**	9.44 ± 0.20 (3)	9.33 ± 0.29 (3)	
**K46A**	9.44 ± 0.20 (3)	9.32 ± 0.24 (3)	
**K47A**	9.54 ± 0.11 (4)	9.31 ± 0.13 (4)	
**M48A**	9.15 ± 0.19 (4)	8.57 ± 0.21 (4)	
**M49A**	9.40 ± 0.24 (3)	9.23 ± 0.22 (3)	
**D50A**	9.22 ± 0.14 (4)	9.13 ± 0.22 (4)	
**Q52A**	9.15 ± 0.18 (5)	9.43 ± 0.19 (5)	
**Y53A**	9.54 ± 0.13 (4)	9.44 ± 0.18 (4)	
**K54A**	9.57 ± 0.14 (3)	9.51 ± 0.29 (3)	
**Y56A**	9.78 ± 0.35 (4)	9.51 ± 0.30 (4)	
**W79A**	9.47 ± 0.22 (5)	7.78 ± 0.22 (5) ***	49
**F99A**	9.61 ± 0.38 (4)	8.83 ± 0.32 (4)	6
**P100Q**	9.94 ± 0.04 (3)	9.84 ± 0.02 (3)	
**D101A**	9.82 ± 0.20 (5)	8.38 ± 0.16 (5) ***	28
**F102A**	9.87 ± 0.17 (5)	7.43 ± 0.21 (5) ***	275
**H121A**	9.51 ± 0.21 (5)	8.76 ± 0.15 (5) *	6
**E123A**	9.60 ±0.11 (4)	9.62 ± 0.07 (4)	
**N124A**	9.60 ± 0.11 (4)	9.40 ± 0.21 (4)	
**N124S**	9.60 ± 0.11 (4)	9.57 ± 0.14 (4)	
**R126A**	9.20 ± 0.23 (4)	9.35 ± 0.37 (4)	
**W128A**	9.64 ± 0.12 (6)	7.71 ± 0.14 (6) ***	85
**Y131A**	9.40 ± 0.36 (4)	8.37 ± 0.30 (4)	11
