# Supplementary material for: An allosteric role for receptor activity-modifying proteins in defining GPCR pharmacology
Source: Cell Discov. 2016 May 17;2:16012–. doi: 10.1038/celldisc.2016.12 (PMC4869360; doi:10.1038/celldisc.2016.12)
Supplement: Supplementary Table S5 [file celldisc201612-s11.pdf]

Supplementary Table S5. Summary of cAMP assay pEC_{50} values for CTR/CLR ECD residue swap mutants at the $CT_{(a)}$ and $AMY_{1(a)}$ ($CT_{(a)}$ /RAMP1) receptors when stimulated with hCT, rAmy or hαCGRP. Data are mean ± SEM. Number of independent experiments indicated in parentheses.

**Mutant**	**hCT**		**rAmy**		**hαCGRP**	
**$CT_{(a)}$**	**pEC_{50} WT**	**pEC_{50} mutant**	**pEC_{50} WT**	**pEC_{50} mutant**	**pEC_{50} WT**	**pEC_{50} mutant**
**G44T**	9.90 ± 0.06 (5)	9.66 ± 0.11 (5)	8.11 ± 0.15 (6)	8.41 ± 0.19 (6)	7.26 ± 0.19 (4)	7.59 ± 0.24 (4)
**P100Q**	9.83 ± 0.09 (4)	9.75 ± 0.15 (4)	8.29 ± 0.14 (4)	8.13 ± 0.22 (4)	-	-
**E123A**	9.88 ± 0.26 (3)	9.90 ± 0.26 (3)	8.34 ± 0.20 (3)	8.38 ± 0.07 (3)	-	-
**N124S**	9.83 ± 0.31 (3)	9.82 ± 0.38 (3)	8.28 ± 0.15 (4)	8.46 ± 0.16 (3)	-	-
**S129T**	9.90 ± 0.19 (4)	9.69 ± 0.27 (4)	8.34 ± 0.20 (3)	8.39 ± 0.08 (3)	-	-
**$AMY_{1(a)}$**						
**G44T**	-	-	9.73 ± 0.18 (5)	9.67 ± 0.11 (5)	9.62 ± 0.15 (5)	9.77 ± 0.16 (5)
**P100Q**	-	-	10.12 ± 0.12 (4)	10.03 ± 0.17 (4)	9.88 ± 0.17 (4)	9.76 ± 0.14 (4)
**E123A**	-	-	9.79 ± 0.14 (4)	9.84 ± 0.17 (4)	9.31 ± 0.26 (5)	9.16 ± 0.14 (5)
**N124S**	-	-	9.69 ± 0.15 (5)	9.64 ± 0.26 (5)	9.08 ± 0.40 (3)	9.54 ± 0.16 (3)
**S129T**	-	-	9.59 ± 0.22 (3)	9.71 ± 0.27 (3)	9.37 ± 0.33 (4)	9.29 ± 0.12 (4)
