# Supplementary material for: An allosteric role for receptor activity-modifying proteins in defining GPCR pharmacology
Source: Cell Discov. 2016 May 17;2:16012–. doi: 10.1038/celldisc.2016.12 (PMC4869360; doi:10.1038/celldisc.2016.12)
Supplement: Supplementary Table S6 [file celldisc201612-s12.pdf]

Supplementary Table S6. Summary of cell surface expression data for RAMP1 ECD mutants at the $AMY_{1(a)}$ receptor. Data are mean ± SEM. Statistical significance (*) was achieved if the 95% confidence does not include 100. The number of independent experiments is indicated in parentheses.

	**$AMY_{1(a)}$ %WT**
**Mutant**	**Anti-myc**
**Y66A**	34.6 ± 5.3 (4) *
**H97A**	76.0 ± 6.5 (4) *
**F101A**	69.3 ± 8.8 (4) *
