# Supplementary material for: An allosteric role for receptor activity-modifying proteins in defining GPCR pharmacology
Source: Cell Discov. 2016 May 17;2:16012–. doi: 10.1038/celldisc.2016.12 (PMC4869360; doi:10.1038/celldisc.2016.12)
Supplement: Supplementary Movie Legend [file celldisc201612-s14.pdf]

Supplemental Movie. Molecular morph between RAMP1/CGRP analog-bound and RAMP2/AM-bound states of the CLR ECD. The movie depicts a morph transitioning back and forth between the CGRP analog-bound CLR:RAMP1 ECD complex (PDB 4RWG) and the AM-bound CLR:RAMP2 ECD complex (PDB 4RWF). The two crystal structures were superimposed based on the CLR positions and Pymol (Schrodinger) was used to generate the morph over a series of 30 frames. Breaks in the RAMP1/2 chain are due to extra amino acids in RAMP1 as compared to RAMP2, which the morph algorithm does not account for. The movie was produced with Pymol.
